# The Relation Between eHealth Literacy and Health-Related Behaviors: Systematic Review and Meta-analysis

**DOI:** 10.2196/40778

**Published:** 2023-01-30

**Authors:** Keonhee Kim, Sangyoon Shin, Seungyeon Kim, Euni Lee

**Affiliations:** 1 Research Institute of Pharmaceutical Sciences College of Pharmacy Seoul National University Seoul Republic of Korea; 2 Department of Pharmacy Seoul National University Bundang Hospital Gyeonggi-do Republic of Korea; 3 College of Pharmacy Dankook University Cheonan Republic of Korea

**Keywords:** eHealth literacy, digital health literacy, online health information, health-related behaviors, health-promoting behavior, meta-analysis

## Abstract

**Background:**

With widespread use of the internet and mobile devices, many people have gained improved access to health-related information online for health promotion and disease management. As the health information acquired online can affect health-related behaviors, health care providers need to take into account how each individual’s online health literacy (eHealth literacy) can affect health-related behaviors.

**Objective:**

To determine whether an individual’s level of eHealth literacy affects actual health-related behaviors, the correlation between eHealth literacy and health-related behaviors was identified in an integrated manner through a systematic literature review and meta-analysis.

**Methods:**

The MEDLINE, Embase, Cochrane, KoreaMed, and Research Information Sharing Service databases were systematically searched for studies published up to March 19, 2021, which suggested the relationship between eHealth literacy and health-related behaviors. Studies were eligible if they were conducted with the general population, presented eHealth literacy according to validated tools, used no specific control condition, and measured health-related behaviors as the outcomes. A meta-analysis was performed on the studies that could be quantitatively synthesized using a random effect model. A pooled correlation coefficient was generated by integrating the correlation coefficients, and the risk of bias was assessed using the modified Newcastle-Ottawa Scale.

**Results:**

Among 1922 eHealth literacy–related papers, 29 studies suggesting an association between eHealth literacy and health-related behaviors were included. All retrieved studies were cross-sectional studies, and most of them used the eHealth Literacy Scale (eHEALS) as a measurement tool for eHealth literacy. Of the 29 studies, 22 presented positive associations between eHealth literacy and health-related behaviors. The meta-analysis was performed on 14 studies that presented the correlation coefficient for the relationship between eHealth literacy and health-related behaviors. When the meta-analysis was conducted by age, morbidity status, and type of health-related behavior, the pooled correlation coefficients were 0.37 (95% CI 0.29-0.44) for older adults (aged ≥65 years), 0.28 (95% CI 0.17-0.39) for individuals with diseases, and 0.36 (95% CI 0.27-0.41) for health-promoting behavior. The overall estimate of the correlation between eHealth literacy and health-related behaviors was 0.31 (95% CI 0.25-0.34), which indicated a moderate correlation between eHealth literacy and health-related behaviors.

**Conclusions:**

Our results of a positive correlation between eHealth literacy and health-related behaviors indicate that eHealth literacy can be a mediator in the process by which health-related information leads to changes in health-related behaviors. Larger-scale studies with stronger validity are needed to evaluate the detailed relationship between the proficiency level of eHealth literacy and health-related behaviors for health promotion in the future.

## Introduction

### Background

The development of digital media and communication technology has increased access to information, and a growing proportion of health-related information is being gained through the internet. A survey conducted in the United States reported that 59% of survey participants had experience in retrieving health information online and 35% had experience in self-diagnosing their health status using online health information [1]. The internet offers the advantage of quick and easy access to a vast amount of up-to-date information and allows communication with health care experts using diverse media platforms such as social networking websites, messengers, and video streaming services [2]. The internet’s capacity goes beyond the realm of merely acquiring health-related information, as bidirectional or multidirectional information sharing is also possible [3].

Furthermore, owing to the widespread penetration of the internet and mobile devices, numerous health care professionals increasingly use web-based or online materials to provide information to patients [4]. Previous studies have demonstrated that digital information can be implemented and used positively for public health projects, such as smoking cessation, weight control, and alcohol addiction management [5-7]. Although access to a wide range of information has improved with the internet, information on the internet comes from a variety of providers and sources that are difficult to control, which can lead to problems with quality and the risk of circulating biased content according to the interests and purposes involved [8].

Therefore, moving forward from the concept of traditional health literacy, the ability to seek, find, understand, and appraise health information from an electronic source has emerged as eHealth literacy [9,10]. Health literacy, a concept preceding eHealth literacy, is shown to be closely associated with health-related factors, such as health behavior, disease management, and quality of life, in various studies [11-13]. Likewise, numerous studies on eHealth literacy, including the development of measurement tools, measurement of individuals’ eHealth literacy, and identification of factors contributing to eHealth literacy, have been steadily conducted [14-17]. However, not much is known about the association between eHealth literacy and health-related behaviors, especially whether eHealth literacy can influence changes in the actual behavior. We raised the question of whether eHealth literacy might be the key mediator from obtaining online health information to changing actual health-related behaviors. Since extensive health information is available online and the acquisition of the information could influence individuals’ health-related behaviors, such as disease management, medication adherence, and seeking health care services [18], a comprehensive review of the influence of eHealth literacy on actual health-related behaviors affected by online health-related information is needed.

### Objectives

Therefore, we performed a systematic review and meta-analysis to describe the effect of eHealth literacy on the types of health-related behaviors and to present the pooled quantitative relationship between them.

## Methods

### Definitions of eHealth Literacy and Health-Related Behaviors

The theoretical definition of eHealth literacy refers to the ability to seek, find, understand, and appraise health information from electronic sources and apply the knowledge gained to addressing or solving a health problem [10]. In our study, eHealth literacy was operationally defined as the total eHealth literacy score measured with a validated measurement tool, such as the eHealth Literacy Scale (eHEALS) developed by Norman and Skinner [19].

Health-related behaviors were defined as “behavioral patterns, actions, and habits that relate to health maintenance, to health restoration, and to health improvement” and included the use of health care services, such as vaccinations and health checkups, compliance with medical therapy, such as treatment diet or medication, and self-directed health behaviors related to diet, exercise, smoking, drinking, etc [20]. In this study, health-related behaviors were operationally defined and subsequently analyzed in the following 3 categories: *health-promoting behavior*, *health-supporting behavior*, and *disease management behavior* (Table 1). *Health-promoting behavior* consisted of the following 6 dimensions: nutrition, physical activity, health responsibility, stress management, interpersonal relations, and self-realization. It was measured with tools such as Health-Promoting Lifestyle Profile (HPLP) II [21]. *Health-supporting behavior* was defined as a health-related behavior that was only a part of the dimension of *health-promoting behavior* or was not included in *health-promoting behavior*. It was measured by the absence or presence of each experience, or the total score obtained using measurement tools of health behaviors such as Healthy Lifestyle and Personal Control Questionnaire [22]. Lastly, *disease management behavior* included any activity performed to manage a specific disease and was quantified by measurement tools according to disease-specific behavioral characteristics such as the Self-Care of Heart Failure Index [23].

**Table 1:** Operational classification and definitions of health-related behaviors.

Health-related behavior	Operational definition
Health-promoting behavior	A holistic behavioral pattern that includes health responsibility, nutrition, physical activity, stress management, interpersonal relations, and self-realization [24]
Health-supporting behavior	Lifestyle habits and disease prevention behaviors for maintaining health [25], which are only a part of the dimension of health-promoting behavior or are not included in health-promoting behavior
Disease management behavior	All activities performed to manage a specific disease

### Literature Search

We performed this systematic review and meta-analysis in accordance with the PICO-SD (Population, Intervention, Comparator, Outcome, Study Design) framework and PRISMA (Preferred Reporting Items for Systematic Reviews and Meta-Analyses) guidelines (Multimedia Appendix 1) [26]. We searched MEDLINE, Embase, Cochrane Central Register of Controlled Trials, KoreaMed, and Research Information Sharing Service to collect research papers published until March 19, 2021. The search keywords combined synonyms of eHealth with synonyms of literacy for a more comprehensive search. Additional manual searches to find relevant studies were also performed by reviewing the bibliographies from the retrieved papers. The detailed search strategy is presented in Multimedia Appendix 2.

### Eligible Criteria and Study Selection

For the systematic review, searched studies were selected according to the following inclusion criteria:

1. Population: Study participants were from the general population and were not health care professionals or students majoring in health care. Participants were not excluded based on their age, race, or morbidity status.
2. Intervention: Studies that reported levels of eHealth literacy measured by validated quantification tools, such as the eHEALS, were included.
3. Comparator: There was no specific comparator.
4. Outcomes: The outcomes of the included studies had to suggest objectively measured health-related behaviors. The behaviors could be evaluated individually or could be integrated.
5. Study design: Studies were selected regardless of their study design, except for qualitative studies.

Studies were excluded if they were (1) not measuring eHealth literacy or not using validated eHealth literacy measurement tools; (2) qualitative studies or not original research papers; (3) not written in either English or Korean; or (4) not available in full text.

The literature search and selection process was performed independently by 2 reviewers (KK and SS). Any discordance among the reviewers during the process of literature selection was resolved through mutual agreement or by involving a third researcher (SK) in a discussion. If two or more studies were performed on the same set of participants, the studies were considered duplicates, and only 1 comprehensive study was selected for further analysis.

### Data Extraction and Risk of Bias Assessment

The following data were extracted from the selected literature using a standardized form by 2 reviewers (KK and SS): the characteristics of the studies (first author, publication year, country or location, study design, participants, and sample size); types of eHealth literacy scales; mean eHealth literacy score; types of health-related behaviors whose correlations with eHealth literacy were verified; methods of measuring health-related behaviors; statistical analysis methods; types of outcome indicators; and values of outcome indicators. Any inconsistency or ambiguity was resolved by discussion with other reviewers (SK and EL).

The risk of bias in the selected studies was assessed using the modified Newcastle-Ottawa Scale (NOS). While the NOS was developed for assessing the risk of bias in nonrandomized observational studies [27], our study used the modified NOS [28] that was developed for cross-sectional studies. The NOS uses a star system to assess the risk of bias in studies, whereby a lower score (ie, number of stars) is associated with a higher risk of bias: high risk, 0-3 stars; unclear risk, 4-6 stars; and low risk, 7-9 stars. Studies evaluated to have a high risk of bias were excluded from the meta-analysis.

### Qualitative and Quantitative Synthesis of the Results

For a qualitative analysis of the results, study country, study population, eHealth literacy measurement tools, types of health-related behaviors and measurement tools, and the relationship between eHealth literacy and health-related behaviors were presented descriptively. The characteristics of the study population were described by age and morbidity status. The specific contents of health-related behaviors were summarized, and they were also classified into the following 3 categories: *health-promoting behavior*, *health-supporting behavior*, and *disease management behavior*. The relationship between eHealth literacy and health-related behaviors was evaluated by whether the effect of eHealth literacy was positive or negative.

For evaluating the association between eHealth literacy and health-related behaviors by a quantitative method, the pooled correlation coefficient was estimated by Fisher z-transformation and construction of the inverse transformation [29]. We used the correlation coefficients of individual studies and treated each result as a separate study when multiple subgroup results were reported in 1 study. The pooled correlation coefficient presented with a 95% CI was tested by performing hypothesis testing to determine whether the correlation was statistically significant. Interpretation of the pooled correlation coefficient was conducted according to Cohen criteria [30]. Cochran Q-statistics and *I^2^*-statistics were used to assess the heterogeneity within the studies included in the meta-analysis, and we applied either the fixed-effects model or random-effects model, depending on the significance of heterogeneity (*P*<.10 and *I^2^*≥50%) [29]. To test the validity of the study results, publication bias was evaluated using a funnel plot and Egger regression, and in case of suspected publication bias, the severity of bias was tested using the trim-and-fill method to estimate the degree to which the publication bias would affect the validity of the study results.

The total effect size (ie, pooled correlation coefficient) was derived from each group of studies divided by the participants’ mean age, morbidity status, and types of health-related behaviors, and from all studies that could be quantitatively synthesized. Through this, we tried to evaluate changes in the effect size according to detailed characteristics. All statistical analyses were performed using Comprehensive Meta-analysis, version 2 software (Biostat).

## Results

### Study Selection

**Figure 1:**
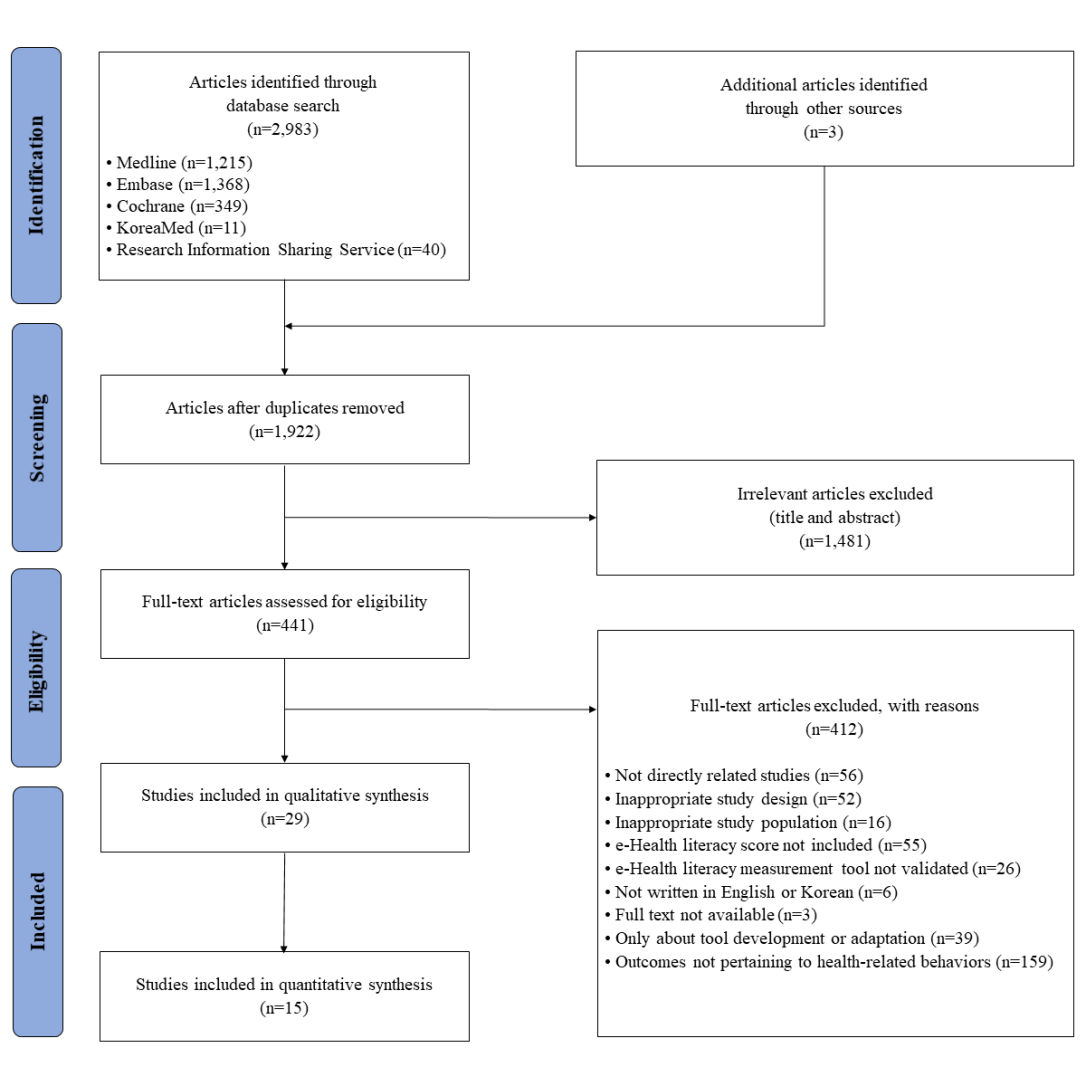
The PRISMA (Preferred Reporting Items for Systematic Reviews and Meta-Analyses) flowchart of the study selection process.

Of 1922 identified nonduplicate studies, 1481 studies were excluded after a review of the studies’ titles and abstracts. The remaining 441 studies were assessed for eligibility through full-text review. Finally, 29 studies, which presented the association between eHealth literacy and health-related behaviors, were selected for qualitative analysis. Out of these, only 14 studies that were quantitatively synthesizable for analysis were included in the meta-analysis. The detailed study selection process with the reasons for exclusion during screening steps is shown in Figure 1.

### Characteristics of the Included Studies

The overall characteristics of the included studies are summarized in Table 2 [31-59]. Among the 29 studies, most were published in South Korea (n=9), followed by Taiwan (n=5), the United States (n=4), China (n=3), Japan (n=3), Turkey (n=2), Germany/Austria (n=1), Iran (n=1), and Pakistan (n=1), showing that most of the studies were conducted in Asia. All retrieved studies were cross-sectional studies using questionnaires. The age groups of the study participants varied, and there were teenagers [31,32], college students [33-40], and older adults [41-46]. While most studies were conducted on the general population regardless of disease status, 6 studies [41,47-51] were conducted on patients with specific diseases, including heart failure, hypertension, diabetes, cancer, and HIV infection.

In 25 out of the 29 studies, the original eHEALS (comprising an 8-item questionnaire with a 5-point Likert scale) or its language or culturally adapted versions for respective countries were used. In the studies analyzed, versions of the eHEALS adapted into Korean, Chinese, Turkish, Japanese, and Persian that were undergoing a reliability test were used. Among the 4 studies that used measurement tools other than the eHEALS, 3 studies [33,39,40] used the 12-item eHealth Literacy Scale (eHLS) and 1 study [35] used a 51-item eHealth Literacy Scale developed and validated in Korean.

Health-related behaviors considered to be correlated with eHealth literacy included *health-promoting behavior*, *health-supporting behavior*, and *disease management behavior*. HPLP Ⅱ was the most frequently used tool for measuring *health-promoting behavior.* The Short-form HPLP, Health-Promoting Lifestyle Scale, and Adolescent Health Promotion Scale were used alongside. *Health-supporting behaviors* included a regular and balanced diet, appropriate physical activity, sufficient sleep, abstinence, smoking cessation, vaccination, safe sex life, prevention of infectious diseases, cancer screening experience, and positive thinking. These behaviors were comprehensively assessed using various tools, including the Health-related Behavior Scale, Healthy Lifestyle and Personal Control Questionnaire, and Dietary Behaviors Scale, as well as self-developed items. *Disease management behaviors* included heart failure self-management, diabetes self-management, chronic disease self-management, and medication adherence. *Disease management behaviors* were measured using validated tools specific to each disease (Table 2).

**Table 2:** Characteristics of the included studies.

Author (year)	Country	Population (sample n)	eHL^a^ measurement tool	Health-related behaviors measurement tool	Types of health-related behaviors	Risk of bias
An et al (2021) [52]	United States	Adults aged over 18 years (n=1074)	Coronavirus-related eHEALS^b^	7 self-reported items	Infection prevention behaviors	6
Blackstock et al (2016) [47]	United States	HIV-infected adult women (n=63)	eHEALS	HIV Risk-Taking Behaviour Scale and Addiction Severity Index	High-risk sexual and drug use behaviors	6
Britt et al (2017) [34]	United States	College students (n=420)	eHEALS	Questions for the 8 health areas identified from the American College Health Association	Diet, exercise, sleep, harmful substances, vaccination, safe sex practices, social relationship, and overall health	3
Cho and Ha (2019) [48]	Korea	Adult outpatients with hypertension (n=156)	Korean version of eHEALS	Self-care behaviors measurement tool	Diet, weight control, stress management, alcohol and tobacco use, physical activity, and medication	7
Choi (2020) [42]	Korea	Older adults aged over 65 years (n=198)	Korean version of eHEALS	Adapted HPLP^c^ Ⅱ	Health responsibility, physical activity, nutrition, spiritual development, interpersonal support, and stress management	6
Chuang et al (2019) [49]	Taiwan	Adults with heart failure (n=141)	Chinese version of eHEALS	22-item instrument Self-Care of Heart Failure Index version 6.2	Self-care maintenance, management, and confidence in heart failure	6
Cui et al (2021) [43]	China	Older adults aged over 60 years (n=1201)	Chinese version of eHEALS	HPLP	Self-actualization, health responsibility, exercise, nutrition, interpersonal support, and stress management	8
Guo et al (2021) [50]	Taiwan	Diabetes mellitus outpatients aged 20 to 65 years (n=249)	eHEALS	36-item Diabetes Self-care Behavior questionnaire	Self-care activities related to diabetes mellitus	4
Gürkan and Ayar (2020) [31]	Turkey	High school students (n=219)	Turkish version of eHEALS	Adolescent Health Promotion Scale	Diet, life appreciation, social support, exercise, stress management, and health responsibility	6
Hsu et al (2014) [33]	Taiwan	College students (n=525)	eHLS^d^	Self-developed 12-item Health Behavior Scale	Diet, exercise, and sleep behaviors	8
Hwang and Kang (2019) [35]	Korea	College students (n=242)	eHL scale composed of functional, communicative, and critical eHL	Adapted HPLP Ⅱ	Health responsibility, physical activity, nutrition, spiritual development, interpersonal support, and stress management	8
Kim and Kim (2020) [51]	Korea	Cancer patients aged 19 to 64 years (n=76)	Adapted eHEALS	Adapted HPLP Ⅱ	Health responsibility, physical activity, nutrition, spiritual development, interpersonal support, and stress management	6
Kim and Son (2017) [53]	Korea	Young adults aged 18 to 39 years (n=230)	Korean version of eHEALS	5-item validated Health-Related Behaviors Scale	Behaviors to prevent disease and promote health	9
Korkmaz Aslan et al (2021) [32]	Turkey	Students aged 14 to 19 years (n=409)	Turkish version of eHEALS	Adolescent Health Promotion Scale	Diet, life appreciation, social support, exercise, stress management, and health responsibility	8
Lee et al (2017) [54]	Korea	Adults aged 20 to 59 years (n=195)	Adapted eHEALS	Adapted HPLP Ⅱ	Health responsibility, physical activity, nutrition, spiritual development, interpersonal support, and stress management	7
Li et al (2021) [44]	China	Older adults aged over 60 years (n=2300)	Chinese version of eHEALS	HPLP	Self-actualization, health responsibility, exercise, nutrition, interpersonal support, and stress management	8
Li and Liu (2020) [55]	China	Internet users aged 20 to 60 years (n=802)	Chinese version of eHEALS	Self-developed 10-item protective behaviors measurement scale	COVID-19 prevention behaviors	7
Lin et al (2020) [41]	Iran	Older adults aged over 65 years with heart failure (n=468)	Persian version of eHEALS	5-item self-reported Medication Adherence Report Scale	Medication adherence	7
Mitsutake et al (2012) [56]	Japan	Adult internet users aged 20 to 59 years (n=2970)	Japanese version of eHEALS	A question with “Yes” or “No” answer	Colorectal cancer screening test	7
Mitsutake et al (2016) [57]	Japan	Internet users aged 20 to 59 years (n=2115)	Japanese version of eHEALS	Self-developed questions	Cigarette smoking, physical exercise, alcohol consumption, sleeping hours, and dietary habits	5
Nam and Jung (2020) [36]	Korea	Korean and Chinese university students (n=240)	Adapted eHEALS	15-item Adapted Health Behavior Scale	Diet, exercise, and sleep behaviors	4
Park et al (2014) [58]	United States	Adults aged over 18 years who had experience using the internet (n=108)	eHEALS	A question with “Yes” or “No” answer	Breast, cervical, colorectal, or prostate cancer screening tests	3
Rabenbauer and Mevenkamp (2021) [59]	Germany and Austria	Facebook users aged over 18 years (n=224)	eHEALS	Healthy lifestyle and personal control questionnaire	Diet, daily time management, physical exercise, social support, and positive thinking	5
Ryu (2019) [45]	Korea	Older adults aged over 65 years (n=99)	Korean version of eHEALS	A tool for measuring the health behavior of elderly people	Diet, exercise, restriction of cigarette smoking or alcohol use, stress management, and disease prevention	7
Song and Shin (2020) [46]	Korea	Older adults aged over 65 years using the internet (n=102)	Korean version of eHEALS	Adapted HPLP Ⅱ	Health responsibility, physical activity, nutrition, spiritual development, interpersonal support, and stress management	8
Tariq et al (2020) [37]	Pakistan	College students (n=505)	eHEALS	Self-developed and validated questions on health behaviors	Physical activity and use of dietary supplements	4
Tsukahara S et al (2020) [38]	Japan	University students (n=3183)	Japanese version of eHEALS	Self-developed questions	Exercise, breakfast, smoking, alcohol consumption, and hours of sleep	6
Yang et al (2017) [39]	Taiwan	College students (n=556)	eHLS	Health-Promoting Lifestyle Scale	Self-actualization, health responsibility, interpersonal support, exercise, nutrition, and stress management	8
Yang et al (2019) [40]	Taiwan	College students (n=813)	eHLS	14-item Dietary Behaviors Scale	Dietary habits	7

^a^eHL: eHealth literacy.

^b^eHEALS: eHealth Literacy Scale.

^c^HPLP: Health-Promoting Lifestyle Profile.

^d^eHLS: 12-item eHealth Literacy Scale.

### Risk of Bias Assessment

The risk of bias assessment revealed that 15 studies had a low risk of bias and 12 studies had an unclear risk of bias (Table 2). Two studies [34,58] were found to have a high risk of bias with a NOS score of 3, and both studies were confirmed to have selection biases, such as representativeness of the study population, calculation of the sample size, and proportion of nonresponders. The high risk of bias in the 2 studies was also attributed to the failure to control confounding variables and to use validated tools for measuring health-related behaviors (Multimedia Appendix 3).

### Qualitative Analysis of eHealth Literacy and Health-Related Behaviors

Among the 29 included studies, 6 showed that not all health-related behaviors were significantly associated with eHealth literacy [36-38,48,51,58] and 1 demonstrated a negative effect of eHealth literacy on health-related behaviors [47]. Significant associations were not found between the eHEALS score and *disease management behaviors* in hypertension patients [48], and between the eHEALS score and *health-promoting behaviors* or *health-supporting behaviors* in cancer patients [51,58] or college students [36,37]. In a study with Japanese university students [38], the eHEALS score was positively associated with some behaviors, such as regular exercise and breakfast eating, but was not associated with behaviors related to sleeping, smoking, and drinking. A study with HIV-infected low-income women [47] identified that higher eHealth literacy was significantly associated with HIV transmission risk behaviors. In the remaining 22 out of 29 studies, positive associations were present between eHealth literacy and health-related behaviors, that is, individuals with higher eHealth literacy scores were reported to show higher scores in *health-promoting behaviors* [31,32,35,39,43,44,46,54], regular eating and exercise [33,34,38,40,42,45,57,59], sufficient sleep [33,34,38,57], stress management [42,45], smoking cessation [34,38,45,57], alcohol abstinence [34,38,45,57], compliance with disease-prevention behaviors [45,52,55,56], medication adherence [41], self-care management of heart failure [49], and self-care management for diabetes [50]. The relationship between eHealth literacy and health-related behaviors in the included studies is summarized in Multimedia Appendix 4.

### Quantitative Analysis of eHealth Literacy and Health-Related Behaviors

#### Correlation Between eHealth Literacy and Health-Related Behaviors by Population Characteristics and Types of Health-Related Behaviors

The correlation coefficients between eHealth literacy and health-related behaviors in the studies [31, 35, 41-43, 45, 46, 48-51, 54, 55, 59] included in the meta-analysis ranged from 0.14 to 0.45. Among these studies, the correlation between eHealth literacy and health-related behaviors was synthesized by age, morbidity status, and types of health-related behaviors. From the age subgroups, the pooled estimates of correlation coefficients were 0.28 (95% CI 0.22-0.34) and 0.37 (95% CI 0.29-0.44) for studies in which participants’ mean age was <65 years and ≥65 years, respectively. While the pooled correlation coefficient of the patient group was estimated at 0.28 (95% CI 0.17-0.39), that of the nonpatient group was 0.32 (95% CI 0.25-0.39). For the subtypes of health-related behaviors, the highest effect size of the pooled correlation coefficient was shown by *health-promoting behavior* (0.36, 95% CI 0.27-0.41), and the lowest was shown by *disease management behavior* (0.24, 95% CI 0.12-0.35) (Table 3).

**Table 3:** Correlation between eHealth literacy and health-related behaviors by age, morbidity status, and type of health-related behavior.

Characteristic			Studies, n		Pooled correlation coefficient, value (95% CI)
**Age**					
	<65 years	9		0.28 (0.22-0.34)	
	≥65 years	5		0.37 (0.29-0.44)	
**Morbidity status**					
	Patients	4		0.28 (0.17-0.39)	
	Nonpatients	10		0.32 (0.25-0.39)	
**Type of health-related behavior**					
	Health-promoting behavior	7		0.36 (0.27-0.41)	
	Health-supporting behavior	3		0.31 (0.19-0.42)	
	Disease management behavior	4		0.24 (0.12-0.35)	

#### Overall Estimate of the Correlation Between eHealth Literacy and Health-Related Behaviors

**Figure 2:**
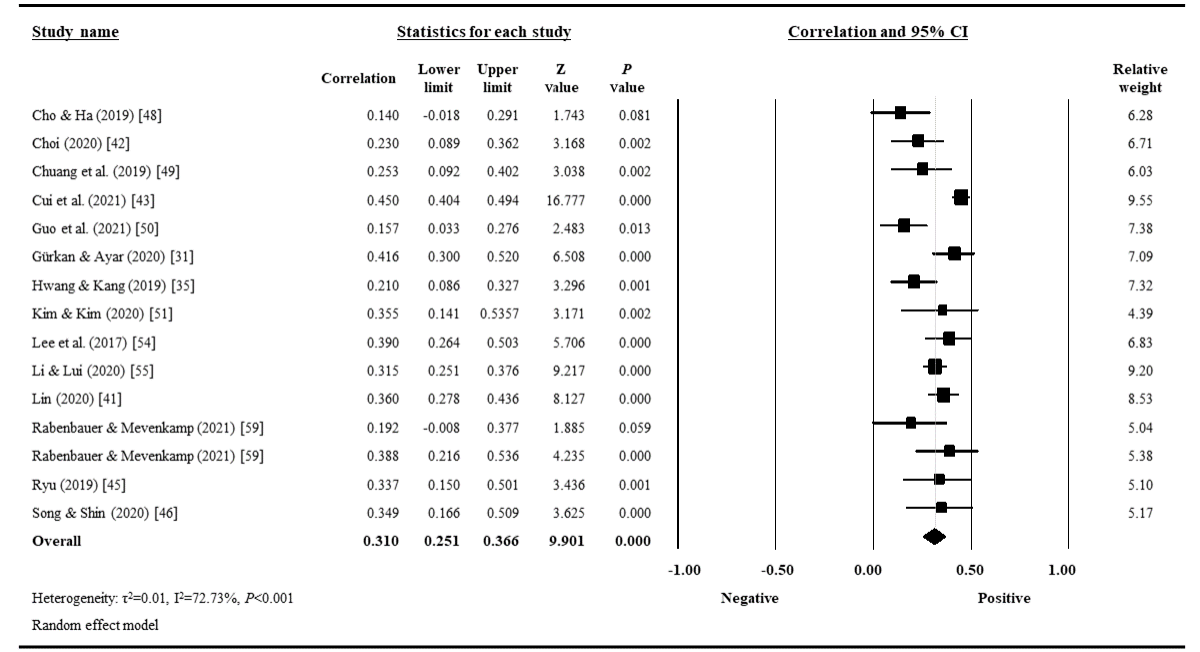
Forest plot of the correlation coefficients between eHealth literacy and health-related behaviors.

The overall estimate of the correlation between eHealth literacy and health-related behaviors was conducted for all 14 studies available for quantitative analysis. The pooled correlation coefficient was 0.31 (95% CI 0.25-0.34; *P*<.001), with high heterogeneity (Cochrane Q=51.34; *P*<.001; *I^2^*=72.73%), which indicated a moderate correlation [30] between eHealth literacy and health-related behaviors (Figure 2).

**Figure 3:**
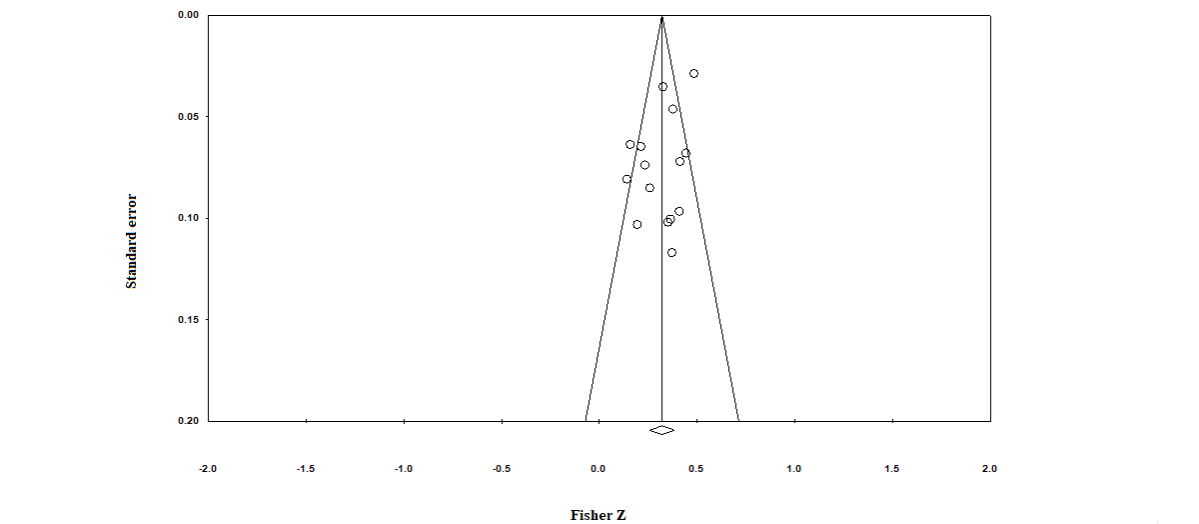
Funnel plot of the correlation between eHealth literacy and health-related behaviors.

Regarding publication bias, slight asymmetry in visual analysis using a funnel plot was observed (Figure 3), and a weak but statistically significant asymmetry was verified in the Egger regression test (*P*=.049). In further trim-and-fill analysis, no studies were found to require further transformation to symmetry, and consequently, no changes in the size effect occurred, indicating that the publication bias did not affect the validity of the study results.

## Discussion

### Prior Work and Principal Findings

The pursuit of health information can affect various health-related outcomes, such as disease prevention actions, perceived health status, and use of health care medical services [60]. With access to and sharing of an enormous amount of health information through the internet in the general population, the concept of eHealth literacy has been studied as a mediator in the process from online health information acquisition to changing individual health-related behaviors [61-63]. Therefore, health care professionals need to have an in-depth understanding of eHealth literacy and its effects on health-related behaviors. In previous studies, concerns were raised that a lower eHealth literacy level was associated with greater difficulty in accessing and understanding online health information, making it more difficult for affected individuals to manage their chronic diseases and to comply with disease prevention actions such as cancer screening [64,65]. However, eHealth literacy may not necessarily have a positive effect on health behaviors or disease-prevention behaviors. A study by Aharony and Goldman [66] found that the group that hesitated to get vaccinated had a higher frequency and intensity of using online information sources. Since eHealth literacy can have a multifaceted effect on health-related behaviors, a more comprehensive analysis is needed to gain an in-depth understanding of the correlation between eHealth literacy and health-related behaviors.

Our meta-analysis showed a moderately positive correlation between eHealth literacy and health-related behaviors, and eHealth literacy was found to have a significant effect on health-related behaviors such as *health-promoting behavior*, *health-supporting behavior*, and *disease management behavior*. However, some of the studies, which specifically focused on younger age groups and were not included in the meta-analysis, showed no association or rather a negative association [36,38,47,58]. Therefore, the effect of eHealth literacy on health-related behaviors should be carefully interpreted, and the possibility of other factors mediating the relationship between eHealth literacy and health-related behaviors should be taken into account. As eHealth literacy alone cannot explain the correlation between online health information acquisition and health-related behaviors, additional research is needed to identify other factors.

In the results of the quantitative analysis by age, the younger population showed a relatively weak correlation between eHealth literacy and health-related behaviors, while the older population showed a moderate correlation. These results might suggest that health-related behaviors in the younger population are influenced by other factors, such as perceived health status and interest in health, going beyond the level of merely obtaining health information [67]. According to previous studies, health literacy among older adults was positively associated with health-related behaviors [68,69], and a computer-based health literacy intervention conducted for elderly people improved participation in their own health care [70]. Considering the results of previous studies and our meta-analysis, eHealth literacy can act as a mediator in changing health-related behaviors using online health information in older adults. Moreover, the older population aged ≥65 years showed a lower level of eHealth literacy than those aged <65 years in previous studies [63,71]. Thus, efforts are needed to promote the level of eHealth literacy in older adults, which is expected to contribute to the promotion of positive health-related behaviors in this population.

The correlation coefficient between eHealth literacy and health-related behaviors was lower in the patient group than in the nonpatient group. Since patients were reported to have more opportunities to obtain health information from various sources, including their doctors or health care providers, when compared with the general population [72], we believe that health-related behaviors showed a relatively low correlation with eHealth literacy in the patient group. The number of patients who find and use health information on the internet is increasing [73-75], and patients’ eHealth literacy can influence their health-related decision-making process and health care provider-patient communication [76,77]. Therefore, the low correlation between eHealth literacy and health-related behaviors should not be interpreted as a finding of overlooking the importance of a patient’s eHealth literacy.

In the quantitative analysis by subtypes of health-related behaviors, a correlation between eHealth literacy and *health-promoting behavior* was most frequently observed (n=7), and its effect size was also the largest. In contrast, a weak correlation was observed between eHealth literacy and *disease management behavior*, and the context was similar to that in the patient subgroup. Therefore, improvement in eHealth literacy is expected to greatly contribute to boosting the level of *health-promoting behaviors* such as eating habits, exercise, stress management, health responsibility, interpersonal relationships, and internal growth. Moreover, it would be beneficial to consider individual eHealth literacy levels when health care professionals provide eHealth services related to health-promoting behaviors.

### Strengths and Limitations

In this study, eHealth literacy–related studies were systematically reviewed and comprehensively summarized. In addition, a pooled effect size was derived for the correlation between eHealth literacy and health-related behaviors using a meta-analysis. This study is significant in that it comprehensively presented the characteristics of the research subjects to be considered for understanding eHealth literacy and provided a resource framework regarding the role of eHealth literacy in health-related behaviors and decision-making. The results of this study suggest that health care providers can manage people’s health behaviors and promote health more effectively by providing eHealth care services that consider individuals’ eHealth literacy. In addition, the moderate correlation between eHealth literacy and health-related behaviors supports the importance of eHealth literacy in the process of health care delivery.

Several limitations of the study should be considered when interpreting the findings. First, the sample size of each study included in the meta-analysis was small, and pooled estimates of the correlation coefficient showed high heterogeneity. Due to this limitation, there is a lack of generalizability, and additional research on eHealth literacy and health behaviors is required to support the results. Second, only studies that provided results in the form of correlation coefficients were included in the meta-analysis. Specifically, studies that presented the relationship between eHealth literacy and health-related behaviors in the form of regression coefficients could not be included in quantitative synthesis to estimate the pooled correlation coefficient. Therefore, the causal relationship between eHealth literacy and health behaviors could not be verified in the study, and further meta-analyses need to be performed on the data to demonstrate the effectiveness of eHealth literacy enhancement programs and the resultant changes in health-related behaviors. Third, the eHealth literacy and health-related behavior measurement items used in the included studies varied, which in turn might have led to biased analysis results. Moreover, the eHealth literacy measurement tools of the included studies, such as eHEALS, were developed in the Web 2.0 era and could not fully assess the concept of Web 3.0. Therefore, it is necessary to consider these points when interpreting the results of this study and applying them to practice. Further studies are needed to better explain the relationship between eHealth literacy and health-related behaviors by using measurement tools that are standardized and appropriate in the Web 3.0 era.

### Conclusion

In this study, a systematic literature review was conducted on the studies investigating the association between eHealth literacy and health-related behaviors, and a meta-analysis was performed on the results of quantitatively synthesizable cross-sectional studies. Our study found that eHealth literacy has fairly significant positive correlations with health-related behaviors such as self-management behavior, medication adherence, disease management, and prevention actions. Among health-related behaviors, *health-promoting behavior* was observed to have the highest correlation with eHealth literacy. The findings from our study indicate that eHealth literacy can be a mediator in the process by which health-related information leads to changes in health-related behaviors. Larger-scale studies with stronger validity are needed to evaluate the detailed relationship between the proficiency level of eHealth literacy and health-related behaviors for health promotion in the future.

## Appendix

Multimedia Appendix 1 PRISMA (Preferred Reporting Items for Systematic Reviews and Meta-Analyses) 2020 checklist.

Multimedia Appendix 2 Search strategy.

Multimedia Appendix 3 Risk of bias assessment for the included studies using the modified Newcastle-Ottawa Scale.

Multimedia Appendix 4 The relationship between eHealth literacy and health-related behaviors in the included studies.

## Abbreviations

eHEALS: eHealth Literacy Scale
HPLP: Health-Promoting Lifestyle Profile
NOS: Newcastle-Ottawa Scale
PRISMA: Preferred Reporting Items for Systematic Reviews and Meta-Analyses
