# Supplementary material for: The Relation Between eHealth Literacy and Health-Related Behaviors: Systematic Review and Meta-analysis
Source: J Med Internet Res. 2023 Jan 30;25:e40778. doi: 10.2196/40778 (PMC9926349; doi:10.2196/40778)
Supplement: Multimedia Appendix 2 [file jmir_v25i1e40778_app2.docx]

**Multimedia Appendix 2.** Search strategy.

| MEDLINE | |
| --- | --- |
| #1 | ((((((((((((((((((("e-Health"[Text Word]) OR ("e-health"[Text Word])) OR ("e health"[Text Word])) OR ("mhealth"[Text Word])) OR ("m-health"[Text Word])) OR ("m health"[Text Word])) OR ("mobile health"[Text Word])) OR ("digital health"[Text Word])) OR ("internet health"[Text Word])) OR ("internet-based health"[Text Word])) OR ("internet based health"[Text Word])) OR ("computer health"[Text Word])) OR ("computer-based health"[Text Word])) OR ("computer based health"[Text Word])) OR ("web health"[Text Word])) OR ("web-based health"[Text Word])) OR ("web based health"[Text Word])) OR ("online health"[Text Word])) OR ("online-based health"[Text Word])) OR ("online based health"[Text Word]) |
| #2 | ((literac*[MeSH Terms]) OR (literac*[Text Word])) OR (literate[Text Word]) |
| #3 | #1 AND #2 |
| EMBASE | |
| #1 | e-Health' OR 'e-health' OR 'e health' OR 'mhealth' OR 'm-health' OR 'm health' OR 'mobile health' OR 'digital health' OR 'internet health' OR 'internet-based health' OR 'internet based health' OR 'computer health' OR 'computer-based health' OR 'computer based health' OR 'web health' OR 'web-based health' OR 'web based health' OR 'online health' OR 'online-based health' OR 'online based health' |
| #2 | literacy'/exp/mj OR literac* OR literate |
| #3 | #1 AND #2 |
| Cochrane | |
| #1 | "e-Health" OR "e-health" OR "e health" OR "mhealth" OR "m-health" OR "m health" OR "mobile health" OR "digital health" OR "internet health" OR "internet-based health" OR "internet based health" OR "computer health" OR "computer-based health" OR "computer based health" OR "web health" OR "web-based health" OR "web based health" OR "online health" OR "online based health" OR "online-based health" |
| #2 | "literacy" OR "literacies" OR "literate" |
| #3 | #1 and #2 in Cochrane Reviews, Trials |
| KoreaMed | |
| #1 | "e-health"[ALL] or "e-Health"[ALL] or "mobile health"[ALL] or "m-health"[ALL] or "mhealth"[ALL] or "digital health"[ALL] or "internet health"[ALL] or "internet-based health"[ALL] or "computer health"[ALL] or "computer-based health"[ALL] or "web health"[ALL] or "web-based health"[ALL] or "online health"[ALL] or "online-based health"[ALL] |
| #2 | "literacy"[ALL] or "literacies"[ALL] or "literate"[ALL] |
| #3 | #1 and #2 |
| RISS | |
| #1 | "e-Health"\|"e-health"\|"e health"\|"mhealth"\|"m-health"\|"m health"\|"mobile health"\|"digital health"\|"internet health"\|"internet-based health"\|"internet based health"\|"computer health"\|"computer-based health"\|"computer based health"\|"web health"\|"web-based health"\|"web based health"\|"online health"\|"online based health"\|"online-based health" |
| #2 | "literacy"\|"literacies"\|"literate" |
| #3 | #1 and #2 |
