# Supplementary material for: The Relation Between eHealth Literacy and Health-Related Behaviors: Systematic Review and Meta-analysis
Source: J Med Internet Res. 2023 Jan 30;25:e40778. doi: 10.2196/40778 (PMC9926349; doi:10.2196/40778)
Supplement: Multimedia Appendix 3 [file jmir_v25i1e40778_app3.docx]

Multimedia Appendix 3. Risk of bias assessment for the included studies using the modified Newcastle-Ottawa Scale.

| **Study (Year)** | **Selection** | | | | | **Comparability** | **Outcome** | | | **Total** |
| --- | --- | --- | --- | --- | --- | --- | --- | --- | --- | --- |
|  | **Representative-ness of the samples^a^** | **Sample size calculation^b^** | **Non-respondents^c^** | | **Use of validated eHL literacy measurement tool^d^** | **Adjustment of confounding factors^e^** | **Use of validated instruments for outcome measurement^f^** | **Ascertainment of the outcome^g^** | **Statistical test^h^** |  |
| An et al (2021) [52] | ★ |  | |  | ★ | ★★ |  | ★ | ★ | 6 |
| Blackstock et al (2016) [47] |  |  | |  | ★ | ★★ | ★ | ★ | ★ | 6 |
| Britt et al (2017) [34] |  |  | |  | ★ |  |  | ★ | ★ | 3 |
| Cho & Ha (2019) [48] |  | ★ | | ★ | ★ | ★★ |  | ★ | ★ | 7 |
| Choi (2020) [42] |  |  | | ★ | ★ | ★ | ★ | ★ | ★ | 6 |
| Chuang et al (2019) [49] | ★ |  | | ★ | ★ |  | ★ | ★ | ★ | 6 |
| Cui et al (2021) [43] | ★ | ★ | | ★ | ★ | ★ | ★ | ★ | ★ | 8 |
| Guo et al (2021) [50] |  |  | | ★ | ★ |  |  | ★ | ★ | 4 |
| Gürkan & Ayar (2020) [31] | ★ | ★ | |  | ★ |  | ★ | ★ | ★ | 6 |
| Hsu et al (2014) [33] | ★ |  | | ★ | ★ | ★★ | ★ | ★ | ★ | 8 |
| Hwang & Kang (2019) [35] |  | ★ | | ★ | ★ | ★★ | ★ | ★ | ★ | 8 |
| Kim & Kim (2020) [51] |  | ★ | | ★ | ★ |  | ★ | ★ | ★ | 6 |
| Kim & Son (2017) [53] | ★ | ★ | | ★ | ★ | ★★ | ★ | ★ | ★ | 9 |
| Korkmaz Aslan et al (2021) [32] | ★ | ★ | |  | ★ | ★★ | ★ | ★ | ★ | 8 |
| Lee et al (2017) [54] |  | ★ | | ★ | ★ | ★ | ★ | ★ | ★ | 7 |
| Li et al (2021) [44] | ★ |  | | ★ | ★ | ★★ | ★ | ★ | ★ | 8 |
| Li & Liu (2020) [55] | ★ |  | | ★ | ★ | ★★ |  | ★ | ★ | 7 |
| Lin et al (2020) [41] | ★ |  | |  | ★ | ★★ | ★ | ★ | ★ | 7 |
| Mitsutake et al (2012) [56] | ★ |  | | ★ | ★ | ★★ |  | ★ | ★ | 7 |
| Mitsutake et al (2016) [57] | ★ |  | |  | ★ | ★ |  | ★ | ★ | 5 |
| Nam & Jung (2020) [36] |  |  | | ★ | ★ |  |  | ★ | ★ | 4 |
| Park et al (2014) [58] |  |  | |  | ★ |  |  | ★ | ★ | 3 |
| Rabenbauer & Mevenkamp (2021) [59] |  |  | |  | ★ | ★★ |  | ★ | ★ | 5 |
| Ryu (2019) [45] |  | ★ | | ★ | ★ | ★★ |  | ★ | ★ | 7 |
| Song & Shin (2020) [46] |  | ★ | | ★ | ★ | ★★ | ★ | ★ | ★ | 8 |
| Tariq et al (2020) [37] |  |  | |  | ★ |  | ★ | ★ | ★ | 4 |
| Tsukahara et al (2020) [38] |  |  | | ★ | ★ | ★★ |  | ★ | ★ | 6 |
| Yang et al (2017) [39] | ★ |  | | ★ | ★ | ★★ | ★ | ★ | ★ | 8 |
| Yang et al (2019) [40] | ★ |  | | ★ | ★ | ★ | ★ | ★ | ★ | 7 |

^a^ Random sampling or somewhat representative of the average in the target population (one star), Selected group or no description of the sampling strategy (no star);

^b^ Performed (one star), Not performed (no star);

^c^ Losses ≤20% (one star), Losses >20% or not reported (no star);

^d^ Yes (one star), No (no star);

^e^ Adjusted by one confounding factor (one star), Adjusted by other confounding factors (one star), Did not control confounding factors (no star), (Maximum two stars);

^f^ Yes (one star), No (no star);

^g^ Primary data (one star), Secondary data or not reported (no star);

^h^ Appropriate statistical test and clearly described including confidence intervals or the probability level (one star), Inappropriate, incomplete, or not described statistical test (no star).
