# Supplementary material for: The Relation Between eHealth Literacy and Health-Related Behaviors: Systematic Review and Meta-analysis
Source: J Med Internet Res. 2023 Jan 30;25:e40778. doi: 10.2196/40778 (PMC9926349; doi:10.2196/40778)
Supplement: Multimedia Appendix 4 [file jmir_v25i1e40778_app4.docx]

**Multimedia Appendix 4.** The relationship between eHealth literacy and health-related behaviors in the included studies.

| **Study (Year)** | **eHealth literacy instrument** | **Types of health-related behaviors** | **Relationship** |
| --- | --- | --- | --- |
| An et al (2021) [52] | Coronavirus-related eHEALS | Adherence to COVID-19 infection prevention behaviors | Individuals with high Coronavirus-related eHEALS scores reported greater participation in protective behaviors than those with low scores. |
| Blackstock et al (2016) [47] | eHEALS | High-risk sexual behavior  High-risk drug use behavior | eHL was negatively associated with HIV prevention behaviors. |
| Britt et al (2017) [34] | eHEALS | Diet, exercise, sleep, harmful substances, vaccination, safe sex practices, social relationship, and overall health | eHL was positively and significantly associated with all 8 areas of health behaviors. |
| Cho & Ha (2019) [48] | Korean version of eHEALS | Diet, weight control, stress management, alcohol and tobacco use, physical activity, and medication | eHL was not significantly correlated with self-care management of hypertension |
| Choi (2020) [42] | Korean version of eHEALS | Health responsibility, physical activity, nutrition, spiritual development, interpersonal support, and stress management | eHL was significantly and positively correlated with health supporting behaviors. |
| Chuang et al (2019) [49] | Chinese version of eHEALS | Self-care maintenance, self-care management, and self-care confidence of heart failure | eHL was positively correlated with self-care management of heart failure. |
| Cui et al (2021) [43] | Chinese version of eHEALS | Self-actualization, health responsibility, exercise, nutrition, interpersonal support, and stress management | eHL was significantly and positively correlated with health promoting behavior. |
| Guo et al (2021) [50] | eHEALS | Self-care activities related to diet, exercise, medication, self-monitoring blood glucose, footcare, and general care | There was a significant correlation between eHL and self-care management of diabetes. |
| Gürkan & Ayar (2020) [31] | Turkish version of eHEALS | Nutrition, life appreciation, social support, exercise, stress management, and health responsibility | There was a positive moderate relationship between eHL and health promoting behavior. |
| Hsu et al (2014) [33] | eHLS | Diet, exercise, and sleep behaviors | Higher level of critical eHL promoted positive health supporting behaviors. |
| Hwang & Kang (2019) [35] | eHL scale composed of functional, communicative, and critical eHL | Health responsibility, physical activity, nutrition, spiritual development, interpersonal support, and stress management | Health promoting behavior had significant and positive relationships with eHL |
| Kim & Kim (2020) [51] | Adapted eHEALS | Health responsibility, physical activity, nutrition, spiritual development, interpersonal support, and stress management | There was no significant correlation between eHL and health promoting behavior |
| Kim & Son (2017) [53] | Korean version of eHEALS | Behaviors to prevent disease and promote health | eHL was the strongest predictor of health supporting behaviors after adjusting other variables. |
| Korkmaz Aslan et al (2021) [32] | Turkish version of eHEALS | Nutrition, life appreciation, social support, exercise, stress management, and health responsibility | eHL significantly and positively predicted all dimensions of health promoting behavior. |
| Lee et al (2017) [54] | Adapted eHEALS | Health responsibility, physical activity, nutrition, spiritual development, interpersonal support, and stress management | There was significant and positive relationship between eHL and health promoting behavior. |
| Li et al (2021) [44] | Chinese version of eHEALS | Self-actualization, health responsibility, exercise, nutrition, interpersonal support, and stress management | eHL was significantly and positively associated with health-promoting behavior. |
| Li & Liu (2020) [55] | Chinese version of eHEALS | Prevention behaviors based on National COVID-19 Protection Manual | Significant and positive correlation existed between eHL and COVID-19 prevention behaviors. |
| Lin et al (2020) [41] | Persian version of eHEALS | Medication adherence for people with chronic illness | There was significantly positive correlation between eHL and medication adherence. |
| Mitsutake et al (2012) [56] | Japanese version of eHEALS | Colorectal cancer screening test | eHL was significantly and positively associated with colorectal cancer screening behavior. |
| Mitsutake et al (2016) [57] | Japanese version of eHEALS | Cigarette smoking, physical exercise, alcohol consumption, sleeping hours, eating breakfast, eating between meals, and balanced nutrition | Individuals with higher eHL scores were more likely to practice the good health supporting behaviors than those with low eHL scores. |
| Nam & Jung (2020) [36] | Adapted eHEALS | Diet, exercise, and sleep behaviors | There was no significant correlation between eHL and health supporting behaviors in Korean students. Negative correlation existed in Chinese students. |
| Park et al (2014) [58] | eHEALS | Breast, cervical, colorectal or prostate cancer screening tests | There was no significant relationship between the level of eHL and prior experience with cancer screening. |
| Rabenbauer & Mevenkamp (2021) [59] | eHEALS | Diet, daily time management, physical exercise, social support, and positive thinking | Significant and positive correlation existed between eHL and health supporting behaviors. |
| Ryu (2019) [45] | Korean version of eHEALS | Nutrition, exercise, restriction of cigarette smoking or alcohol use, stress management, and disease prevention | eHL had a positive correlation with health supporting behaviors. |
| Song & Shin (2020) [46] | Korean version of eHEALS | Health responsibility, physical activity, nutrition, spiritual development, interpersonal support, and stress management | eHL was positively correlated with health promoting behavior. |
| Tariq et al (2020) [37] | eHEALS | Frequency of moderate physical activity, and use of dietary supplements | eHL was not associated with health supporting behaviors such as physical activity and dietary supplement intake. |
| Tsukahara et al (2020) [38] | Japanese version of eHEALS | Exercise, breakfast, smoking, alcohol consumption, and hours of sleep | Higher eHL was associated with regular exercise and breakfast eating. Sufficient sleep, smoking, and alcohol consumption were not associated with eHL. |
| Yang et al (2017) [39] | eHLS | Self-actualization, health responsibility, interpersonal support, exercise, nutrition, and stress management | eHL, especially critical eHL was positively associated with health promoting behavior. |
| Yang et al (2019) [40] | eHLS | Dietary habits | High eHL was related to positive dietary behaviors. |
